# Dynamics of SOX2 and CDX2 Expression in Barrett's Mucosa

**DOI:** 10.1155/2016/1532791

**Published:** 2016-09-27

**Authors:** Rita Barros, Daniela Pereira, Catarina Callé, Vânia Camilo, Ana Isabel Cunha, Leonor David, Raquel Almeida, António Dias-Pereira, Paula Chaves

**Affiliations:** ^1^Institute of Molecular Pathology and Immunology of the University of Porto (IPATIMUP), Porto, Portugal; ^2^Instituto de Investigação e Inovação em Saúde (i3S), Porto, Portugal; ^3^Portuguese Oncology Institute Francisco Gentil, EPE, Lisbon, Portugal; ^4^Beira Interior University, Covilhã, Portugal; ^5^Faculty of Medicine of the University of Porto, Porto, Portugal; ^6^Biology Department, Faculty of Sciences of the University of Porto, Porto, Portugal

## Abstract

Barrett's esophagus (BE) is the replacement of the normal esophageal squamous epithelium by a columnar lining epithelium. It is a premalignant condition for the development of adenocarcinoma of the esophagus and esophagogastric junction. BE is associated with gastroesophageal reflux which might change the expression profile of key transcription factors involved in the establishment of tissue differentiation, namely, SOX2 (associated with esophageal and gastric differentiation) and CDX2 (associated with intestinal differentiation). Here, we sought to characterize the expression profile of SOX2 and CDX2 in the sequential alterations of the esophageal mucosa towards adenocarcinoma and compare it with the well-established gastric and intestinal mucin profiles (MUC5AC, MUC6, and MUC2). We observed that SOX2 and CDX2 expression correlates with gastric and intestinal differentiation in BE, defined by morphological parameters and mucin expression. We show the presence of a complete intestinal profile in BE, without gastric mucins and without SOX2, and we observed an evolutionary modulation of the metaplastic phenotype by SOX2 and CDX2. We observed that adenocarcinomas harbor more frequently a mixed gastric and intestinal phenotype. In conclusion, our study establishes a role for transcription factors SOX2 and CDX2 in the progression from gastric to gastrointestinal differentiation in Barrett's metaplasia.

## 1. Introduction

Barrett's esophagus (BE), the replacement of the normal esophageal squamous epithelium by a columnar lining that predisposes to cancer [1], is the premalignant condition for the development of adenocarcinoma (ADC) of the esophagus and esophagogastric junction [2, 3]. Barrett, in his first description [4], stressed the presence of the gastric type columnar lining and later Bremner et al. [5] demonstrated its acquired nature and the role of the gastroesophageal reflux in its biopathogenesis. Later on, Paull et al. [6] demonstrated the presence of three distinct types of epithelia in the metaplastic segments exhibiting gastric and intestinal features. The discussion on the phenotypic characteristics of the columnar esophageal lining began in the second half of the last century and reached our days. Actually, during the last decades a lot of work has been done on the differentiation of Barrett's epithelium [7–14]. Presently, evidence suggests that the esophageal columnar lining has a mixed gastric and intestinal phenotype distributed as a mosaic on a gradient, according to the pH gradient (an increased amount of goblet cells in the proximal part of the esophagus) [15]. It is also clear that the metaplastic columnar esophageal lining is not phenotypically stable but, on the contrary, it evolves through time. Initially, it shows a gastric (cardiac type) mucosa with mucous columnar cells that progresses over time to an intestinal-type mucosa, harboring columnar nongoblet and goblet cells with normal and abnormal/aberrant differentiation [16–20].

Presently, it is consensual that Barrett's metaplastic epithelium contains a mixture of cell lineages with gastric and intestinal features fulfilling the definition of incomplete metaplasia [21]. The presence of intestinal metaplasia (IM) is not consensual for the diagnosis of BE [22, 23] but it has a recognized potential for malignant transformation/progression [2, 23].

Tissue differentiation is controlled by transcription factors with restricted expression patterns that become aberrantly expressed in lesions harboring abnormal differentiation [24]. It is expected that the evolutionary phenotypic change observed in Barrett's epithelium involves alterations in these proteins. Although the mechanism is not fully elucidated, it is possible that the metaplastic microenvironment, namely, the reflux pH gradient, alters the transcription factor expression profile of stem cells, leading to the production of cell types characteristic of a different tissue [24]. Thus, the balance between different transcription factors, such as those involved in intestinal and esophageal differentiation (CDX2 and SOX2, resp.), may play a key role in the onset and maintenance of Barrett's epithelium [11, 25].

CDX2 is a homeobox transcription factor critical for intestinal differentiation, in normal conditions only expressed in intestinal epithelium [26]. CDX2 becomes* de novo* expressed throughout the gastrointestinal tract in lesions with intestinal differentiation, such as BE and gastric intestinal metaplasia [11, 25, 27]. It is well demonstrated, in mouse models, that Cdx2 alone is sufficient to induce metaplastic transformation of the gastric mucosa [28]. Different studies, using animal models, have shown that CDX2 expression may be induced by bile acids, present in the gastroesophageal reflux, leading to differentiation reprogramming of squamous epithelium to a glandular intestinal one [29, 30].

SOX2 was identified as a critical transcription factor for esophageal differentiation (but also trachea and lung), both during embryogenesis and in the adulthood [31, 32]. SOX2 is a sex-determining region Y-box 2 gene, a member of the high mobility group (HMG) domain proteins, that is essential to maintain pluripotency in embryonic stem (ES) cells and also to reprogram fibroblasts into induced pluripotent cells (iPS) [33, 34]. In mice, it has also been identified as an adult stem cell marker, in different tissues [35]. Genomic studies have shown that lung and esophagus squamous cell carcinomas (SCC) frequently harbor SOX2 gene amplification and aberrant expression levels [36].

Here, we sought to characterize SOX2 and CDX2 expression in the distinct morphological steps recognized on the evolutionary phenotype of Barrett's metaplasia and in Barrett's ADC (BA), in an attempt to evaluate their involvement in the differentiation reprogramming of human esophageal mucosa.

## 2. Material and Methods

### 2.1. Samples

We studied retrospectively 10 patients with initial diagnosis of columnar-lined esophagus (CLES) ≥2 cm, defined by the absence of IM in the two first endoscopies (performed with at least 1-year interval); 18 cases of BE defined by the presence of columnar epithelium with IM areas, 8 of which were adjacent to ADC (BEadj.ADC) and nonintestinal (gastric type) epithelium being present in 9 of the 18 BE cases; 4 dysplasia cases; and 8 cases of BA. All cases were selected from the BE surveillance program of Instituto Português de Oncologia de Lisboa, which started in 1992 [37]. We also took into account the clinical evolution of the patients (CLES and BE), by analyzing subsequent biopsies from the same patients, when available, to search for progression markers. The use of retrospective samples from which informed consent cannot be obtained is authorized for research studies by the Portuguese law.

### 2.2. Immunohistochemistry

Paraffin-embedded specimens were subjected to immunohistochemistry for the transcription factors SOX2 and CDX2 and mucins MUC5AC, MUC6, and MUC2, using the antibodies and conditions described in Table 1. Detection of CDX2, MUC5AC, and MUC2 was performed by incubation with a biotin-labeled rabbit anti-mouse secondary antibody (DAKO, 1 : 100) followed by incubation with an avidin/biotin detection system (Vectastain ABC kit, Vector Laboratories, Burlingame, CA, USA) and development with 3,3′-diaminobenzidine (DAB) (Sigma). Detection of SOX2 was done using the DAKO REAL™ Envision™ Detection System Peroxidase/DAB+ (DAKO, Glostrup, Denmark) according to the manufacturer's instructions.

For SOX2/MUC5AC double staining, immunohistochemistry was first performed for SOX2 using the DAKO REAL Envision Detection System Peroxidase/DAB+ (DAKO, Glostrup, Denmark) immediately followed by immunohistochemistry for MUC5AC using the Envision G2 System/AP (Permanent Red) (DAKO), according to the manufacturer's instructions. Tissue counterstaining was performed using Mayer hematoxylin.

## 3. Results

### 3.1. SOX2, CDX2, and Mucin Expression in CLES and BE

Results regarding SOX2 and CDX2 expression are summarized in Table 2.

**Figure 1:**
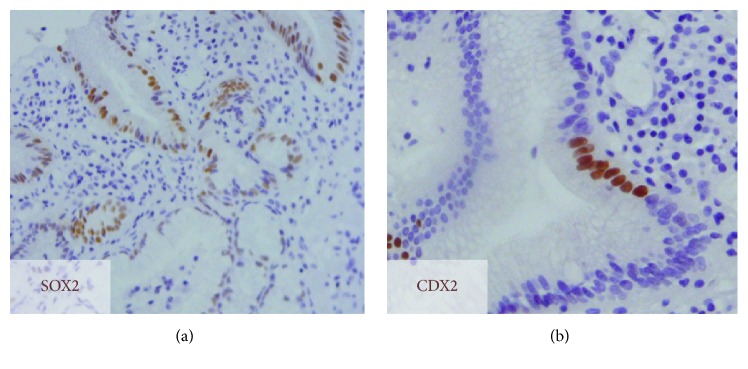
SOX2 and CDX2 expression in columnar-lined epithelial segments (CLES). Immunodetection of (a) SOX2 and (b) CDX2, showing widespread and focal staining (brown), respectively.

**Figure 2:**
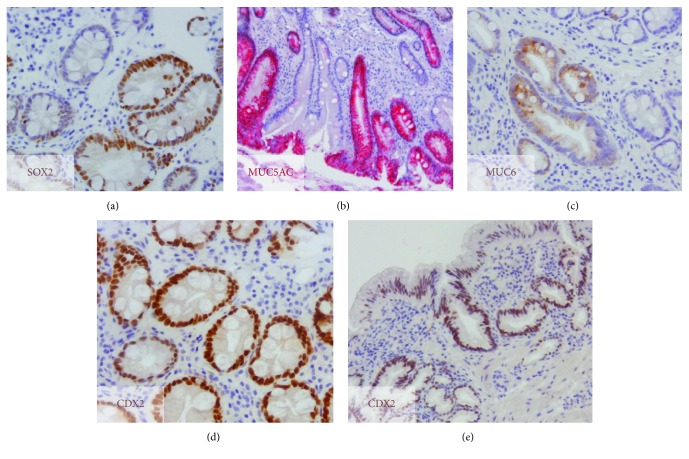
Expression of gastric and intestinal markers in BE lesions. (a) Heterogeneous SOX2 expression; (b) immunostaining for MUC5AC (red) and (c) for MUC6 (brown) in IM areas; (d) homogeneous CDX2 immunostaining in IM glands; (e) CDX2 immunostaining (brown) of a gastric area of BE.

In CLES, SOX2 nuclear expression was observed in all the cases (Figure 1(a)). On the other hand, CDX2 was only expressed in one CLES (1/10), focally and in few cells (Figure 1(b)). In IM areas of all the BE cases, SOX2 expression was heterogeneous (18/18), with negative and positive IM glands (Figure 2(a)). To confirm the mixed gastric and intestinal phenotype, we performed a complementary study with gastric mucins MUC5AC and MUC6 and intestinal mucin MUC2 in a subset of cases (7/18). In most IM glands, SOX2 expression was associated with positivity for at least one gastric mucin (Figures 2(b) and 2(c)), suggestive of an association with the gastric phenotype. On the other hand, CDX2 was expressed throughout (Figure 2(d)) in IM areas (18/18). One exception was a case of BE adjacent to ADC, in which CDX2 was negative. In 9 BE cases (4 adjacent to ADC), gastric metaplasia was present adjacent to IM glands. In these areas, SOX2 was expressed throughout, whereas CDX2 was observed in 5 cases (2 adjacent to ADC) (Figure 2(e)).

### 3.2. Evolution of CLES Cases in Subsequent Endoscopies

**Figure 3:**
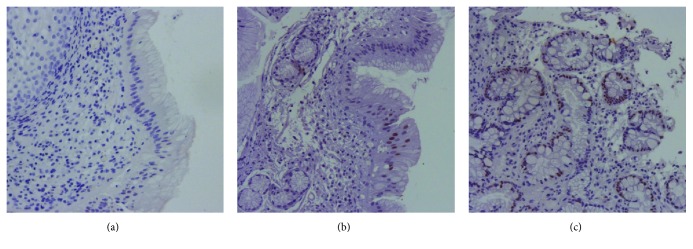
CDX2 expression in single-patient subsequent biopsies. (a) CDX2 negative immunostaining in the index biopsy with CLES, (b) a subsequent biopsy (4 years later) with CLES showing focal CDX2 positivity, and (c) CDX2 expression in BE (9 years after index biopsy) with extensive CDX2 positivity.

From the 10 CLES cases, 5 progressed (developed IM), after a period of 3 to 9 years (median evolution time was 4 years). The case where CDX2 expression was detected already in CLES did progress to IM after 4 years, accompanied by increased CDX2 expression over time. This case was also positive for MUC5AC, MUC6, and MUC2 in CLES (data not shown). Interestingly, one of the initial CLES negative for CDX2 in the index biopsy (Figure 3(a)) was focally positive for CDX2 in a subsequent biopsy (Figure 3(b)) with absence of IM and progressed to IM after 9 years of the first biopsy (and 5 years from the intermediate biopsy) (Figure 3(c)).

### 3.3. SOX2 and CDX2 Expression in Dysplasia and BA

**Figure 4:**
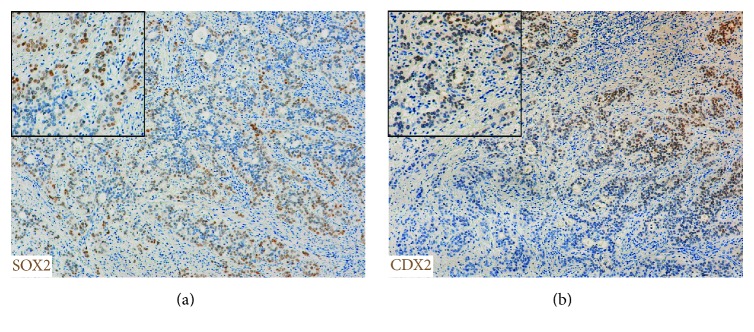
SOX2 and CDX2 expression in esophageal adenocarcinoma. (a) Heterogeneous SOX2 and (b) CDX2 immunostaining (brown). Inserts show higher magnifications.

Results regarding SOX2 and CDX2 expression are summarized in Table 2. The 4 dysplastic lesions studied exhibited different combinations of these transcription factors: 2 cases were positive for both transcription factors (SOX2 was expressed throughout while CDX2 was focal), 1 case was SOX2 positive and CDX2 negative and 1 case was SOX2 negative and CDX2 positive. In BA, SOX2 was detected in all the 8 cases while CDX2 was negative in 1 case. SOX2 and CDX2 were coexpressed in the cells' nuclei of 7 cases, presenting an heterogeneous expression pattern, with positive and negative patches (Figures 4(a) and 4(b)).

## 4. Discussion

In this study we describe the expression of two transcription factors, CDX2 and SOX2, in a series of esophageal columnar metaplasia without IM (CLES) and with IM as well as in dysplasia and BA. The results obtained are strongly indicative of a transcription factor evolutionary phenotype accompanying the well-defined morphological alterations of Barrett's metaplasia. For the first time, we have studied the evolution pattern of these transcription factors from the beginning, the CLES cases. Fifty percent of our CLES (5/10) progressed (developed IM) in subsequent biopsies (after 3 to 9 years), and one (that progressed 4 years later) already had expression of the intestinal marker CDX2 in the CLES index biopsy. However, the small number of cases neither allow speculation about the probability of progression, from CLES to BE, nor establish a time correlation between the intestinal immunophenotype and the development of IM. Nonetheless, it suggests that CDX2 could constitute an early marker of progression to overt IM. CDX2 as an early marker of intestinal reprogramming has been previously explored both in esophageal and gastric settings. In the stomach, CDX2 expression was observed in cells without intestinal differentiation, in a Portuguese and Mozambican population, but correlation with progression to IM could not be established, at least in part due to short follow-up [38]. In esophagus, this phenotype has been previously described but whether it is indicative of a higher risk of progression to BE remains to be clarified [11, 13]. Reinforcing a role of CDX2 in this carcinogenic pathway, we observed a high percentage of CDX2 positivity in dysplasia and BA. This is in agreement with previous observations in the gastric setting [39].

Barrett's metaplasia is a preneoplastic condition that predisposes to cancer. One of the key questions while monitoring patients with BE is the identification of those that have higher risk of developing cancer. Most patients with BE will never develop cancer [1, 2] but they will anyhow be followed up for many years, which has associated health care systems' financial charges and personal costs. It is essential to identify new biomarkers that complement the characterization of Barrett's lesions and might contribute to risk stratification. Barrett's metaplasia follows a sequential alteration in the differentiation profile that evolves from an initial step of gastric differentiation to a mixed gastrointestinal differentiation profile [8, 9].

The present study profiles the evolutionary coordination of key differentiation transcription factors, SOX2 and CDX2, together with the expression of classical gastric and intestinal mucin markers. Within BE we identified glands with “pure” intestinal differentiation, exhibiting expression of intestinal mucins and the transcription factor CDX2, and loss of all gastric markers, including the mucins MUC5AC and MUC6 and the transcription factor SOX2. This is the first study to associate loss of SOX2 with progression to “pure” intestinal differentiation in human BE. However, Chen et al. [40] had previously described absence of SOX2 expression in IM in a rat model of BE. In the gastric mucosa, two subtypes of IM have been clearly identified based on loss or persistence of gastric differentiation together with acquisition of the intestinal one, identified by the expression of mucin markers; complete IM only expresses intestinal mucins, whereas incomplete IM expresses both gastric and intestinal mucins [39, 41]. Incomplete IM is associated with increased potential to evolve to more aggressive lesions [41]. It is tempting to speculate that complete IM also occurs in Barrett's metaplasia and is the final differentiation stage after evolution from a gastric and a mixed gastrointestinal phenotype.

Our study also shows that esophageal adenocarcinomas express both gastric and intestinal markers, suggesting that a mixed gastric/intestinal aberrant differentiation is more prone to progress to malignancy. In rare cases it may follow a nonintestinal pathway for cancer, as proposed by Brown et al., a few years ago. According to this author, there are initial BA, surrounded by cardiac-type mucosa, not associated with IM [42]. This was recently reinforced in the study by Lavery et al., where columnar metaplasia without goblet cells was shown to be the precursor of BA, using lineage tracing [43]. In contrast, van Olphen and colleagues showed that loss of SOX2 is associated with progression to neoplastic lesions in the context of Barrett's metaplasia and that more than 60% of the BA did not express SOX2 [44].

## 5. Conclusion

In conclusion, our study establishes a role for the transcription factors SOX2 and CDX2 in the progression from gastric to gastrointestinal differentiation in Barrett's metaplasia. Moreover, it emphasizes the early phenotypic modulation of the metaplastic epithelium promoted by the two transcription factors suggesting a multistep reprogramming pathway towards malignancy.

## Figures and Tables

**Table 1:** Primary antibodies and immunohistochemistry conditions used in this study.

Antibody	Clone	Antigen retrieval buffer	Antigen retrieval conditions	Dilution	Incubation time (min)	Localization	Source
CDX2	CDX2-88	Citrate buffer 10 mM pH 6.0	40 minutes at 98°C	1 : 50	Overnight (4°C)	Nuclear	Biogenex, San Ramon, CA
MUC2	PMH1	0.1 U/mL neuraminidase^*∗*^	2 h at 37°C	Undiluted	Overnight (4°C)	Cytoplasmatic	Supernatant [28]
MUC5AC	CLH2	None	None	1 : 10	Overnight (4°C)	Cytoplasmatic	Supernatant [28]
MUC6	CLH5	None	None	1 : 10	Overnight (4°C)	Cytoplasmatic	Supernatant [28]
SOX2	SP-76	EDTA 10 mM pH 8.0	40 minutes at 98°C	1 : 50	1 h (room temperature)	Nuclear	Cell Marque, Rockling, CA

^*∗*^Neuraminidase from *Clostridium perfringens* type VI (Sigma) was diluted in sodium acetate buffer (pH 5.5).

**Table 2:** Summary of the results regarding SOX2 and CDX2 expression.

	SOX2		CDX2	
	Positive	Negative	Positive	Negative
CLES (*n* = 10)	10	0	1	9
BE (*n* = 10)				
Intestinal areas (*n* = 10)	10	0	10	0
Gastric areas (*n* = 5)	5	0	3	2
BEadj.ADC (*n* = 8)				
Intestinal areas (*n* = 8)	8	0	7	1
Gastric areas (*n* = 4)	4	0	2	2
Dysplasia (*n* = 4)	3	1	3	1
BA (*n* = 8)	8	0	7	1
